# A novel TNFR2 agonist peptide for the expansion of CD4+Foxp3+ regulatory T cells

**DOI:** 10.3389/fimmu.2025.1696587

**Published:** 2025-11-26

**Authors:** Ping Liao, Zhonghao Chen, Yang Gao, Yang Yang, Yibo Chen, Jiamin Chen, Yiru Wang, Chon-Kit Chou, Shaoyi Zhang, Xin Chen

**Affiliations:** 1 Institute of Chinese Medical Sciences, State Key Laboratory of Mechanism and Quality of Chinese Medicine, University of Macau, Taipa, Macau SAR, China; 2 Department of Pharmaceutical Sciences, Faculty of Health Sciences, University of Macau, Taipa, Macau SAR, China; 3 Ministry of Education (MoE) Frontiers Science Center for Precision Oncology, University of Macau, Taipa, Macau SAR, China

**Keywords:** TNFR2, CD4+Foxp3+ regulatory T cells, peptide, immunosuppression, inflammation

## Abstract

CD4^+^Foxp3^+^ regulatory T cells (Tregs) are essential for maintaining immune tolerance, and selective expansion of Tregs via TNFR2 signaling represents a promising therapeutic approach for autoimmune and inflammatory diseases. Here, we report the identification and characterization of UMR2-705, a novel TNFR2 agonist discovered through phage display screening. *In vitro*, peptide UMR2–705 selectively promoted Treg proliferation in both human peripheral blood mononuclear cells and murine CD4^+^ T cell cultures without stimulating conventional CD4^+^ effector or CD8^+^ T cells. This effect was abrogated by the TNFR2-specific blocking antibody TR75-54.7, indicating its TNFR2 dependency. *In vivo*, administration of peptide UMR2–705 expanded Tregs in murine spleen and lymph nodes, attenuated LPS-induced systemic cytokine release (IL-6, TNF-α, IL-17A) in serum, and preserved immune homeostasis during systemic inflammation through TNFR2-dependent modulation of the regulatory compartment. Transcriptomic profiling revealed activation of TNFR2-associated signaling and upregulation of immune-regulatory pathways. These findings identify peptide UMR2–705 as a selective, peptide-based TNFR2 agonist with potent Treg-expanding and anti-inflammatory activities, supporting its potential as a therapeutic candidate for autoimmune and inflammatory disorders.

## Introduction

CD4^+^Foxp3^+^ regulatory T cells (Tregs) are immunosuppressive cells that play a critical role in maintaining immune homeostasis and preventing autoimmune diseases, including allergic diseases, graft-versus-host disease (GVHD), and transplant rejection (1, 2). Modulating Treg activity holds significant therapeutic promise; for instance, enhancing Treg function can effectively attenuate autoimmune and inflammatory responses (3). Accordingly, targeting the molecular pathways that regulate Treg function offers a strategic approach for immune intervention across diverse disease settings. In 2007, we first reported that tumor necrosis factor (TNF) promotes the activation and expansion of Tregs through its receptor TNFR2 (4). TNF selectively upregulates TNFR2 expression on Tregs, and the TNF-TNFR2 interaction is dispensable for their *in vivo* function and phenotypic stability (5, 6). Since then, extensive evidence has reinforced the pivotal role of TNFR2 signaling in Treg activation, expansion, and stability in response to TNF stimulation (7–9). Consequently, TNFR2 has emerged as a promising therapeutic target, with current translational research efforts focused on developing biologics and small molecules to modulate TNFR2 activity in autoimmune diseases, GVHD, and cancer.

Several TNFR2 agonists have been shown to suppress inflammatory responses by enhancing Treg function and expansion (10–14). TNFR2 agonism provides an effective strategy for expanding low-purity human Tregs, thereby supporting its application in adoptive Treg transfer therapies. Combining TNFR2 agonistic antibodies with standard Treg expansion protocols (e.g., anti-CD3/CD28 stimulation, IL-2, with or without rapamycin) generates stable, homogeneous Tregs with robust immunosuppressive function (15, 16). TNFR2 agonism holds promise for treating autoimmunity due to its restricted expression on immunosuppressive cells like Tregs. This contrasts with low-dose IL-2 therapy, which expands Tregs but also activates unwanted effector cells, such as Teff and NK cells, due to its narrow therapeutic window, thereby increasing the risk of off-target immune activation and related adverse effects (17, 18). The restricted expression of TNFR2 suggests a potentially more targeted therapeutic strategy (19, 20). To enhance TNFR2 signaling, dodecavalent ligands engineered using the oligomerization domain from GCN4 and TNFR2-selective TNF mutants (GCN4-sc-mTNFR2) have demonstrated superior bioactivity and binding affinity *in vitro* compared to other oligomerized TNFR2-selective TNF variants (21). Additionally, GCN4-sc-mTNFR2 may be less immunogenic due to its structural resemblance to human proteins, although immunogenicity remains a concern given its non-natural sequences. It is also important to distinguish our strategy of TNFR2 agonism from the well-established anti-TNF therapies, such as etanercept and infliximab, which act by broadly neutralizing TNF. While these biologics are highly effective in treating various autoimmune diseases through inhibition of the proinflammatory signaling predominantly mediated by TNFR1, they concurrently suppress the beneficial immunoregulatory effects via TNFR2. Such non-selective blockade of TNF may underlie some of the known limitations of anti-TNF therapy, including an increased susceptibility to infections and potential disruption of immune homeostasis and tissue repair (22). In contrast, our approach favors selectively potentiatinge TNFR2 signaling. Such non-selective blockade of TNF may underlie some of the known limitations of anti-TNF therapy, including an increased susceptibility to infections and potential disruption of immune homeostasis and tissue repair (22). In contrast, our approach seeks to selectively potentiate TNFR2 signaling. By employing a TNFR2-specific agonist, we aim to harness the intrinsic immunosuppressive and tissue-protective functions of the TNF–TNFR2 axis, thereby offering a more targeted therapeutic strategy that mitigates the drawbacks associated with global TNF inhibition. Compared with conventional biologics, peptides offer distinct advantages, including lower production costs, facile synthesis, and reduced immunogenicity (23).

In this study, we aimed to develop a novel peptide that targets the TNF-TNFR2 interaction as a selective Treg stimulator. Through phage display screening, we identified peptide UMR2-705 (also referred to as 705), which promotes Tregs expansion without affecting CD8^+^ T cells or conventional effector T cells (Teffs), acting through TNFR2 signaling. These findings highlight the potential of TNFR2-targeting peptides as immunomodulatory agents for the treatment of autoimmune and inflammatory diseases.

## Results

### Peptide UMR2–705 increased the stimulatory effect of TNF on Treg cells

**Figure 1:**
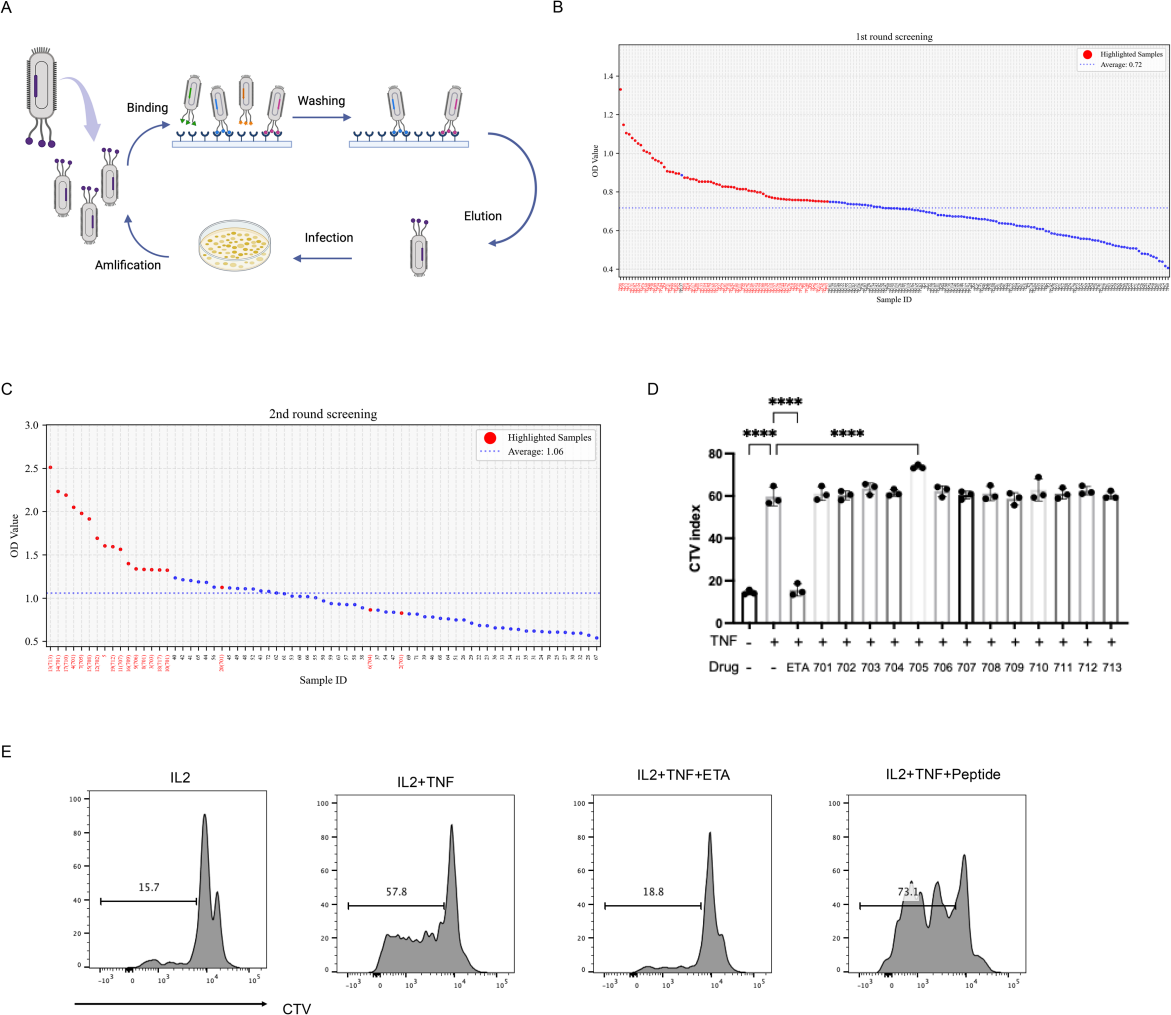
Screening for potent TNFR2 agonist peptide for Treg expansion by phage display. **(A)** Schematic illustration of phage display peptide library screening. **(B)** Dot plot of ELISA OD values showing 72 high-affinity phage clones (red dot). **(C)** Dot plot of ELISA OD values showing 20 top clones (red dot) selected from the 72 high-affinity phage clones. **(D)** Mixed T cells isolated from mouse spleen and lymph nodes were labeled with CellTrace™ Violet (CTV) and cultured for 72 hours with IL-2 (10 ng/mL) alone, or in combination with TNF (20 ng/mL) and one of 13 different peptides (5 µg/mL). Quantification of Treg proliferation across treatment groups. **(E)** Treg cell proliferation was assessed by CTV dilution and analyzed via flow cytometry. ****P < 0.0001 by one-way ANOVA tests. Figure1A Created in BioRender. Chen, Z. (2025) https://BioRender.com/jzy9iea.

To identify peptides that bind to TNFR2, three rounds of biopanning were performed using a phage display library. Following selection, individual phage clones were randomly isolated, and sequencing revealed 20 unique nucleotide sequences (**Figures 1A-C**). The corresponding amino acid sequences of enriched clones are listed in **Supplementary Table 1**. Based on these results, 13 representative peptides were synthesized for further evaluation. We previously demonstrated that TNF preferentially promotes the proliferation and expansion of Tregs, along with increased surface expression of TNFR2 on these cells (4, 24). In the current study, mixed T cells cultured with interleukin-2 (IL-2) and treated with peptide UMR2–705 showed a significant enhancement of TNF-induced Treg proliferation. In contrast, the TNF inhibitor etanercept (ETA) markedly suppressed TNF-induced Treg expansion (**Figures 1D, E**). Overall, our findings support the conclusion that peptide UMR2–705 functions as an agonist that enhances TNF-induced Treg expansion.

### Peptide UMR2–705 induces dose-dependent expansion of Tregs in murine CD4^+^ T cells and human PBMCs

**Figure 2:**
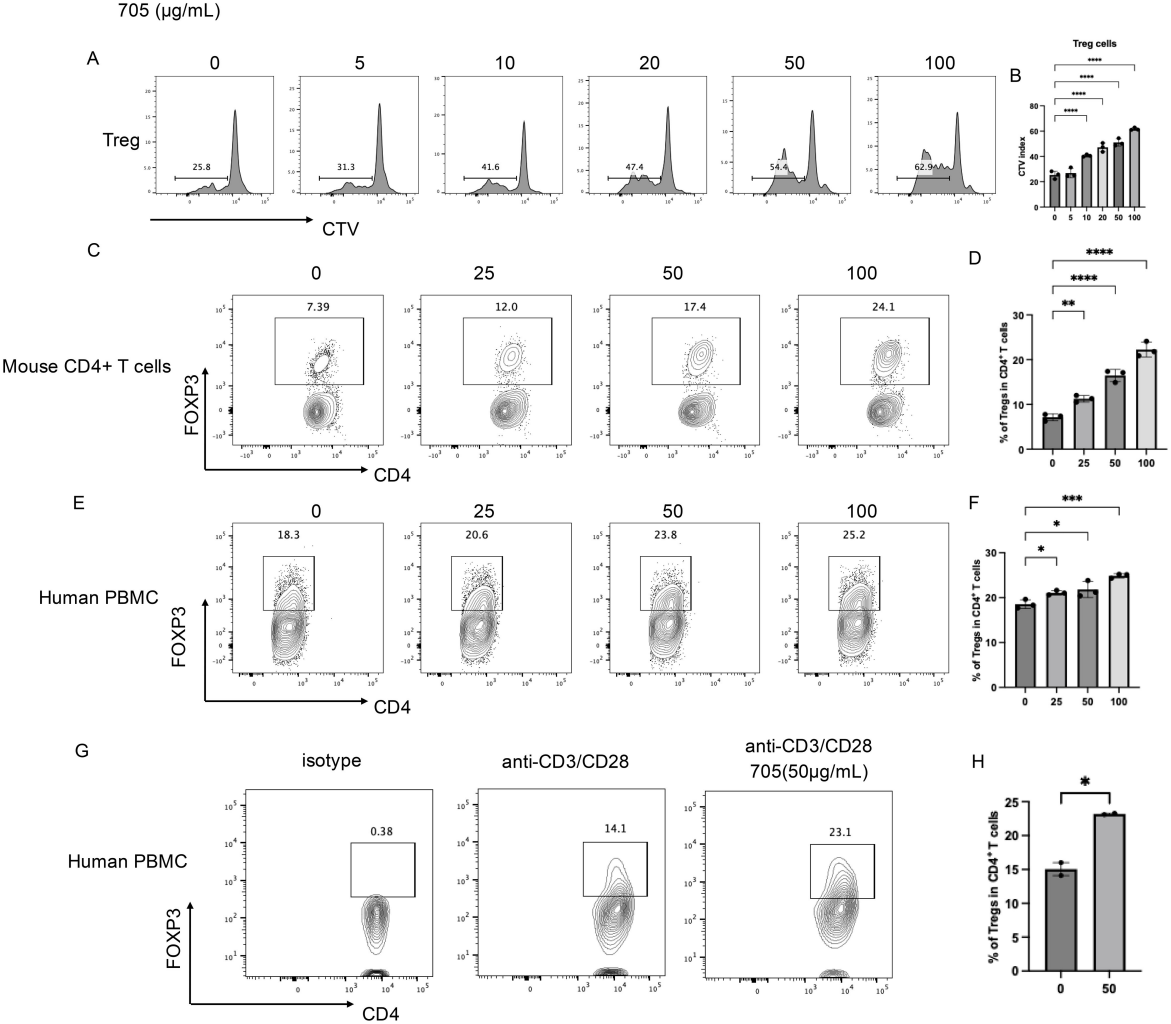
*In vitro* pharmaceutical Evaluation of peptide UMR2–705 in Treg activation.(**A, B**) Peptide UMR2–705 promotes Treg proliferation in murine mixed T cell cultures. **(A)** Representative FACS plots of proliferating CD4^+^FOXP3^+^ Tregs. **(B)** Quantification of proliferative responses in Treg cells. **(C, D)** Peptide UMR2–705 dose-dependently increases Treg frequency in purified murine CD4^+^ T cells. **(C)** Representative flow plots (gated on CD4^+^ T cells). **(D)** Quantification of Treg proportions among total CD4^+^ T cells. **(E, F)** Peptide UMR2–705 elevates Treg frequency in human PBMCs. **(E)** Representative flow plots (gated on CD4^+^ T cells). **(F)** Quantification of Treg frequencies in human CD4^+^ T cells. **(G, H)** Peptide UMR2–705 enhances Treg frequency in anti-CD3/CD28-activated human PBMCs. **(G)** Representative flow plots (gated on CD4^+^ T cells). **(H)** Quantification of Treg frequencies in activated human CD4^+^ T cells. Data are representative of or quantified from at least three independent experiments (n = 3). Statistical significance was determined by one-way ANOVA; *P < 0.05, **P < 0.01, ***P < 0.001, ****P < 0.0001.

To evaluate the effect of peptide UMR2–705 on T cells, splenic lymphocytes were cultured *in vitro* with increasing concentrations of peptide UMR2-705 (0, 5, 10, 20, 50, and 100 µg/mL) and analyzed for proliferation after 72 hours using flow cytometry. Peptide UMR2–705 promoted a dose-dependent induction of Treg proliferation (**Figure 2A, B**).

To furtherly assess the ability of peptide UMR2–705 to expand Tregs, we evaluated its effects on both murine CD4^+^ T cells and human peripheral blood mononuclear cells (PBMCs) *in vitro*. MACS-purified murine CD4^+^ T cells were treated with increasing concentrations of peptide UMR2–705 for 72 hours. Flow cytometric analysis revealed a dose-dependent increase in the proportion of CD4^+^FOXP3^+^ Tregs compared to untreated controls (**Figures 2C, D**). These CD4^+^FOXP3^+^ Tregs are shown to have a stronger suppressive transcriptional signature, including elevated gene expression of Icos, Ikzf2, Ctla4, and Il10 (**Supplementary Figure S1A–D**).

Similarly, treatment of human PBMCs with peptide UMR2–705 resulted in a significant, concentration-dependent elevation in the frequency of CD4^+^FOXP3^+^ Tregs (**Figures 2E, F**). To confirm its effect on human Treg proliferation, MACS-purified CD4+ T cells from human PBMCs were activated with anti-CD3/CD28 antibodies for 48 hours. Treatment with peptide UMR2–705 significantly increased the frequency of regulatory T cells (Tregs) compared with the activated control (**Figures 2G, H**). These results indicate that peptide UMR2–705 effectively promotes the selective expansion of Tregs across both murine and human immune cell populations.

### Peptide UMR2–705 selectively promotes Treg expansion through TNFR2 signaling

**Figure 3:**
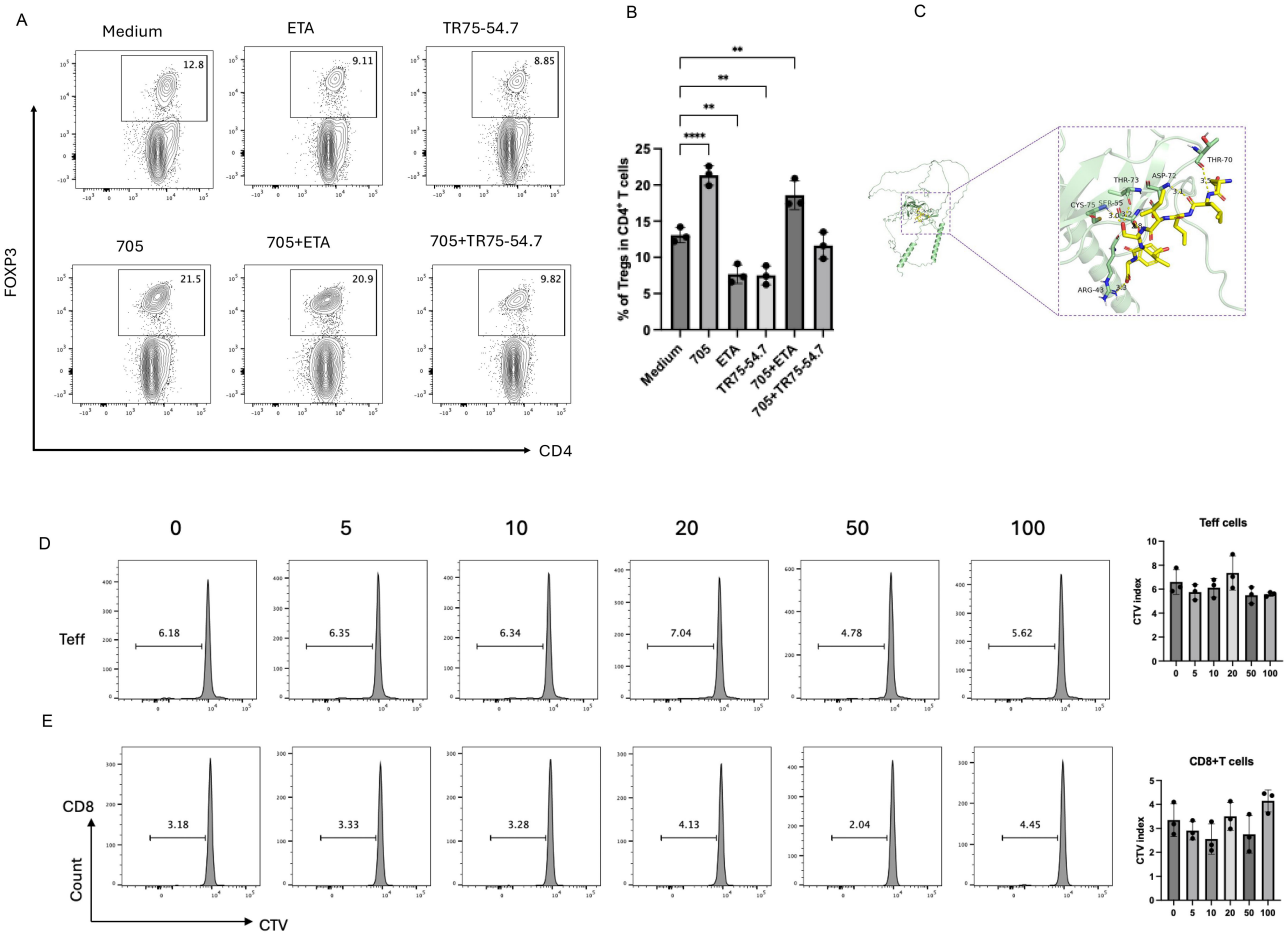
Peptide UMR2–705 selectively activates Tregs via TNF-TNFR2 signaling. **(A, B)** Anti-TNFR2 antibody (TR75-54.7) blocks peptide UMR2-705-induced Treg proliferation in MACS-purified murine CD4^+^ T cells. **(A)** Representative flow plots (gated on CD4^+^ T cells) and **(B)** quantification of CD4^+^Foxp3^+^ Tregs after 72h culture. **(C)** A detailed view of the interaction interface between peptide UMR2–705 and TNFR2. Key residues on TNFR2 involved in hydrogen bonding are shown as sticks. Hydrogen bonds are indicated by yellow dashed lines. **(D, E)** Peptide UMR2–705 dose-dependently inhibits proliferation in mixed T cell cultures. Representative FACS plots of proliferating cells and quantification in CD4^+^FOXP3^−^ effector T cells (Teffs) and **(D)** CD8^+^ T cells **(E)**. Data are representative of at least three independent experiments and are presented as mean ± SEM. Statistical significance was determined using one-way ANOVA; **P < 0.01, ****P < 0.0001.

To determine whether the Treg-expanding activity of peptide UMR2–705 is mediated through TNFR2 signaling, murine CD4^+^ T cells were cultured with peptide UMR2–705 in the presence or absence of TR75-54.7, a TNFR2-specific blocking antibody. Flow cytometric analysis demonstrated that peptide UMR2–705 significantly increased the frequency of Tregs, whereas co-treatment with TR75-54.7 effectively abolished this expansion (**Figures 3A, B**). This result demonstrates that the Treg-promoting effect of peptide UMR2–705 is strictly dependent on TNFR2 engagement.

Molecular docking of peptide UMR2–705 to TNFR2 predicted a strong interaction, with a calculated binding energy of -5.3 kcal/mol. The binding was stabilized by a network of hydrogen bonds to TNFR2 residues ARG-43, CYS-75, SER-55, THR-73, ASP-72, and THR-70. Notably, the predicted binding site of UMR2–705 substantially overlaps with the native binding site of TNF (25). These results provide a structural model for the specific binding of peptide UMR2–705 to TNFR2 and suggest a mechanism of action via receptor agonism.

In addition, in TNFR2-overexpressing Jurkat cells(JK-R2), treatment with biotin-UMR2–705 followed by detection with APC-conjugated streptavidin revealed strong binding of biotin-UMR2–705 to Jurkat-R2 cells, whereas no binding was observed in control Jurkat-NC cells (**Supplementary Figure S2A**).

We next assessed whether the effect of peptide UMR2–705 was specific to Tregs. Splenic lymphocytes were cultured *in vitro* with increasing concentrations of peptide UMR2-705 (0, 5, 10, 20, 50, and 100 µg/mL) and assessed by CTV dilution. Notably, peptide UMR2–705 did not induce proliferation of CD4^+^Foxp3^−^ effector T cells (Teff) or CD8^+^ T cells (**Figures 3D–E**). Together, these results indicate that peptide UMR2–705 selectively expands Tregs in a TNFR2-dependent manner, without stimulating the proliferation of other T cell subsets.

### *In vivo* administration of peptide UMR2–705 increases the proportion of Tregs in the spleen and lymph nodes

**Figure 4:**
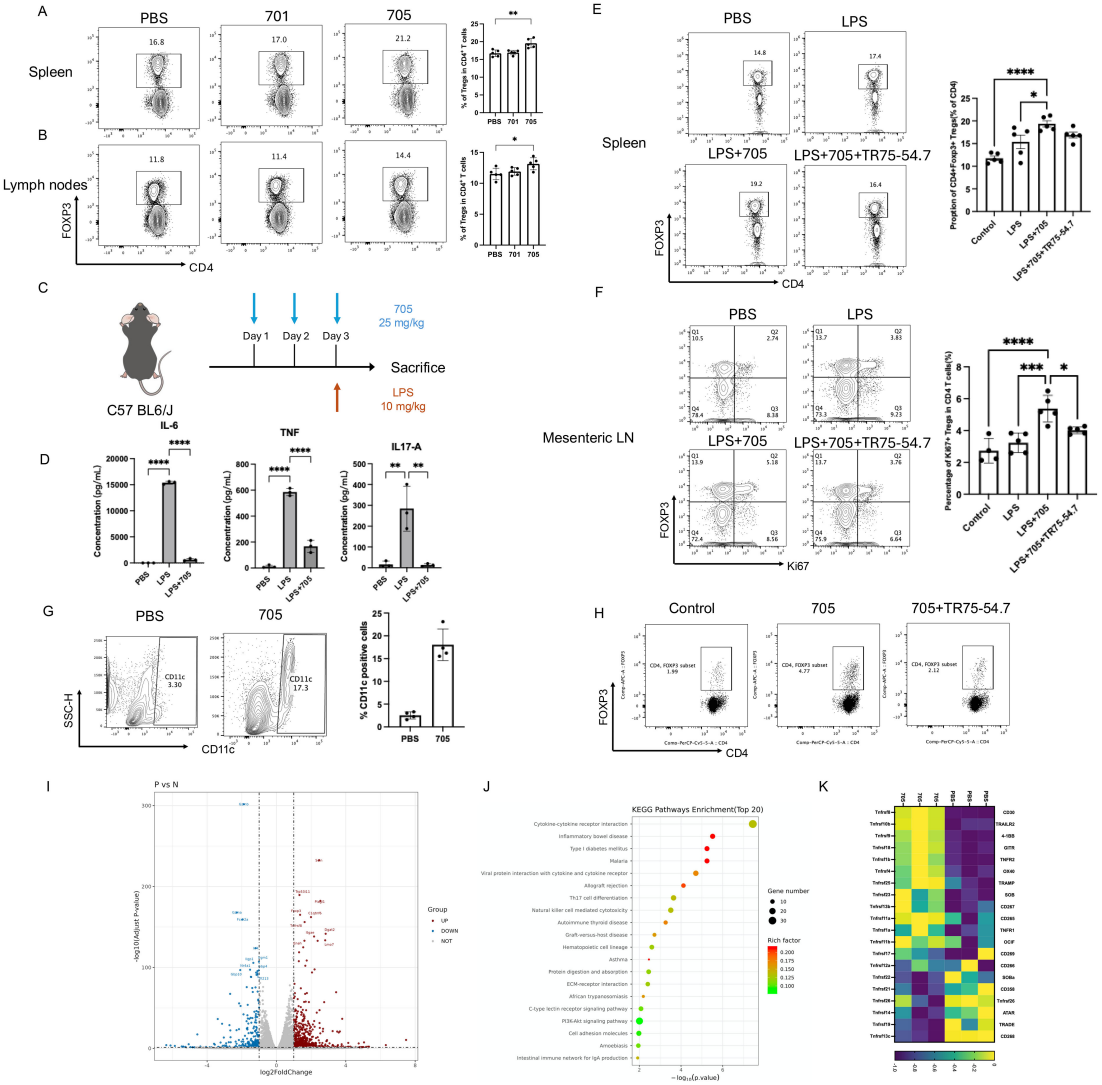
Peptide UMR2–705 increases the proportion of Treg cells in the spleen and lymph nodes of Balb/c mice. **(A, B)** Increased splenic **(A)** and lymph node **(B)** CD4^+^Foxp3^+^ Treg frequency in mice treated i.p. with Peptide UMR2-705 (25 mg/kg/day x 3 days) vs PBS. Representative flow plots (gated on CD4^+^ T cells) and quantification are shown. **(C–F)** Peptide UMR2-705 (25 mg/kg/day x 3 days, i.p.) attenuates LPS (10 mg/kg)-induced inflammation with or without TNFR2-antagonist TR75-54.7(10mg/kg). **(D)** Serum cytokine levels (CBA). **(E)** CD4^+^Foxp3^+^ Treg proportion in spleen. **(F)** Proliferating CD4^+^Foxp3^+^ Treg cells in mesenteric lymph nodes (mLN). **(G)** Increased CD11c^+^ DC frequency in peritoneal lavage fluid of Peptide UMR2-705-treated mice (i.p.). Representative plots and quantification. **(H)** Peptide UMR2-705-preconditioned BMDCs enhance Treg proliferation *in vitro* (co-culture with CD4^+^ T cells). Representative flow plots (gated on CD4^+^ T cells) and quantification of CD4^+^Foxp3^+^ cells. **(I–K)** Transcriptome analysis of CD4^+^ T cells treated with Peptide UMR2-705 (72h). **(K)** Volcano plot of DEGs (|log2FC| > Y, adj. p < Z). **(L)** Enriched KEGG pathways in upregulated DEGs. **(M)** Relative mRNA expression of TNF receptor superfamily genes. Data are presented as mean ± SEM from at least three independent experiments (n = 3). Statistical significance was determined using one-way ANOVA; *P < 0.05, **P < 0.01, ***P < 0.001, ****P < 0.0001.

To evaluate the *in vivo* immunomodulatory effect of peptide UMR2-705, Balb/c mice were intraperitoneally injected with PBS, peptide UMR2-705, or control peptide 701 for three consecutive days. Twenty-four hours after the final treatment, cells from the spleen and lymph nodes were harvested and analyzed by flow cytometry to assess the frequency of CD4^+^Foxp3^+^ regulatory T cells (Tregs). Peptide UMR2–705 treatment significantly increased the proportion of Tregs in both the spleen and lymph nodes compared to PBS or control peptide 701 (**Figures 4A, B**). These findings demonstrate that peptide UMR2–705 promotes the expansion or accumulation of Tregs *in vivo*, supporting its potential as a therapeutic agent for modulating immune responses.

### Peptide UMR2–705 mitigates LPS-induced systemic inflammation and cytokine production

To assess the anti-inflammatory efficacy of peptide UMR2-705, we first profiled a murine model of LPS-induced systemic inflammation. Mice were pretreated with PBS or UMR2-705 (25 mg/kg/day) for three consecutive days prior to a single intraperitoneal injection of LPS (**Figure 4C**). Twenty-four hours later, serum cytokine levels were quantified by cytometric bead array (CBA). As expected, LPS challenge markedly elevated circulating IL-6, TNF, and IL-17A levels compared with PBS controls. Pretreatment with UMR2–705 substantially attenuated this cytokine surge, indicating a pronounced anti-inflammatory effect (**Figure 4D**).

To determine whether UMR2–705 influences immune regulation, we analyzed Treg populations in the spleen and mesenteric lymph nodes. LPS exposure led to a moderate expansion of Tregs, reflecting a compensatory response to acute inflammation. Remarkably, UMR2–705 treatment further increased the proportion of Tregs in the spleen, with a more pronounced rise in Ki67^+^ Tregs among CD4^+^ T cells observed in the mesenteric lymph nodes (mLN), indicating enhanced proliferation and activation of the regulatory compartment (**Figures 4E, F**). To further evaluate whether the expansion of Tregs induced by UMR2–705 is dependent on TNFR2 signaling, mice were co-administered the TNFR2 antagonist TR75-54.7 from the first day of treatment. TNFR2 blockade completely abolished the UMR2-705–induced augmentation of Tregs, confirming that the immunoregulatory activity of UMR2–705 is mediated through TNFR2 engagement.

Together, these findings demonstrate that peptide UMR2–705 alleviates LPS-induced systemic inflammation by promoting TNFR2-dependent expansion of Tregs, thereby reinforcing immune tolerance *in vivo.*

### Peptide UMR2–705 promotes dendritic cell accumulation *in vivo* and enhances their capacity to expand regulatory T cells *in vitro*

In addition to its direct effect on Tregs, we observed that intraperitoneal administration of peptide UMR2-705 (25 mg/kg/day for three consecutive days) markedly increased the frequency of CD11c^+^ dendritic cells (DCs) in the peritoneal lavage fluid of treated mice (**Figure 4G**). To further investigate the functional relevance of these DCs, bone marrow–derived dendritic cells (BMDCs) were generated from mice, treated with peptide UMR2-705, and subsequently subjected to maturation stimuli. These DCs were then co-cultured with MACS-purified CD4^+^ T cells isolated from mouse spleens. Strikingly, peptide UMR2-705–treated DCs significantly upregulate the proportion of CD4^+^Foxp3^+^ Tregs in the co-culture system compared with untreated controls (**Figure 4H**).

These findings suggest that, beyond directly promoting Treg expansion, peptide UMR2–705 may also indirectly augment Treg proliferation by modulating DC function, thereby revealing an additional mechanism of its immunoregulatory activity.

### Peptide UMR2–705 activates inflammation-associated signaling pathways

To investigate the molecular effects of peptide UMR2–705 on TNF receptor signaling, transcriptomic profiling was performed on purified CD4^+^ T cells treated with either PBS or peptide UMR2–705 for three days. RNA sequencing revealed substantial transcriptional alterations in response to peptide UMR2-705, with 573 genes significantly upregulated and 286 genes downregulated (**Figure 4J**). Among the most upregulated genes were Foxp4, Tnfrsf8, and C1qtnf6, while downregulated genes included Gzmb, Irf4, and Nr4a1, etc. KEGG pathway enrichment analysis demonstrated significant activation of immune-related pathways, notably cytokine-cytokine receptor interaction, inflammatory bowel disease, and type I diabetes mellitus (**Figure 4J**). In particular, peptide UMR2–705 treatment led to increased expression of multiple members of the TNF receptor superfamily, including Tnfrsf10b (TRAILR2), Tnfrsf9 (4-1BB), Tnfrsf18 (GITR), Tnfrsf1b (TNFR2), and Tnfrsf4 (OX40), suggesting enhanced TNFR2-mediated signaling activity (**Figure 4K**). Previous studies have shown that TNFR2 signaling correlates with the expression of 4-1BB, GITR, and OX40 in Tregs, and that TNFR2 co-stimulation augments their surface levels, indicating coordinated regulation within the TNFRSF network (26). Moreover, co-expression and functional synergy among TNFR2, 4-1BB and GITR, support a shared regulatory axis that promotes Treg proliferation and survival (27, 28). This coordinated upregulation pattern further indicates that peptide UMR2-705–treated Tregs exhibit an enhanced functional phenotype consistent with heightened TNFRSF signaling activity. Collectively, these findings indicate that peptide UMR2–705 modulates key transcriptional programs associated with immune regulation and underscores its therapeutic potential in promoting Treg-mediated immunomodulation.

## Discussion

The TNF-TNFR2 signaling axis has emerged as a promising target in immunotherapy, with relevance to both cancer treatment and the management of autoimmune diseases (19, 29). TNFR2 is expressed at high levels on Tregs and plays a pivotal role in their expansion and functional maintenance, making it a compelling candidate for immunomodulation strategies (4, 6, 30). In this study, we employed phage display screening of a random peptide library and identified peptide UMR2-705, which selectively expanded Tregs without affecting conventional CD4^+^ effector T cells (Teffs) or CD8^+^ T cells.

Functional validation in both murine and human peripheral blood mononuclear cell (PBMC) models confirmed that peptide UMR2–705 robustly promotes the expansion of Tregs. Importantly, this effect was abrogated by TNFR2-specific antagonists TR75-54.7 antibody both *in vivo* and *in vitro*, confirming that peptide UMR2–705 functions as a TNFR2 agonist. Beyond its immunoregulatory activity, peptide UMR2–705 also exhibited anti-inflammatory properties in an *in vivo* model of LPS-induced systemic inflammation, significantly attenuating the elevation of pro-inflammatory cytokines such as IL-6, TNF, and IL-17A. Together, these findings support the dual functionality of peptide UMR2–705 as both a Treg-expanding agent and an anti-inflammatory modulator, with potential therapeutic relevance for autoimmune and inflammatory disorders.

Parallel to our findings, there is a growing interest in developing TNFR2-targeting agents for clinical use (19, 29). Several TNFR2-targeting antibodies are currently under clinical evaluation, including antagonists such as BI-1808 (Phase 1/2a, NCT04752826), being tested alone or in combination with anti-PD-1 therapy (Merck) (31), as well as APX601 (32), AN3025 (33), and NBL-020 (34). Agonistic antibodies in development include BI-1910 (35), HFB200301 (36), and MM-401 (37). Despite the encouraging progress in antibody-based therapeutics, peptide-based approaches remain largely underexplored. Peptides offer several potential advantages, including lower immunogenicity, cost-effective synthesis, and improved tissue penetration due to their smaller molecular size (38). Our study introduces peptide UMR2–705 as a novel TNFR2-targeted agent with robust immunomodulatory activity. These results underscore the therapeutic promise of peptide-based TNFR2 agonists as either standalone agents or complementary alternatives to existing antibody-based strategies.

The role of TNFR2 signaling in modulating immune responses is complex and context-dependent, particularly in the settings of autoimmune diseases and cancer. Mechanistically, TNFR2 engagement may influence a diverse range of immune cell subsets, including regulatory T cells (Tregs) (4, 6), myeloid-derived suppressor cells (MDSCs) (22, 39, 40), conventional CD4^+^ T cells (Tcon) (41, 42), CD8^+^ T cells (43, 44), and may also contribute to antibody-dependent cellular cytotoxicity (ADCC) (43). While TNFR2 signaling is essential for the stability and suppressive function of Tregs (16, 19, 39), its activation can also enhance effector T cell responses, particularly in CD8^+^ T cells (45). Thus, the therapeutic outcome of TNFR2 modulation is likely determined by the nature of the agonist, the disease context, and tissue-specific immune dynamics. In this regard, peptide UMR2–705 demonstrated selective activity toward Tregs without detectable effects on CD4+Teffs or CD8^+^ T cells, suggesting a more targeted approach to immunomodulation that minimizes pro-inflammatory risk.

This study has several limitations that also highlight valuable directions for future investigation. A key consideration lies in its translational relevance to human disease. While our findings in mouse models provide a strong mechanistic foundation and proof-of-concept for TNFR2 activation by peptide UMR2-705, further validation using human primary immune cells or humanized mouse models will be necessary to establish direct applicability to human physiology. In addition, the pharmacokinetic characteristics of peptide UMR2–705 *in vivo* remain to be fully elucidated. To address this critical aspect, future work will focus on characterizing the peptide’s metabolic stability and clearance *in vivo*. If its stability proves insufficient for therapeutic use, established peptide optimization strategies—such as D-amino acid substitution or site-specific glycosylation—will be applied to enhance metabolic resistance and improve pharmacokinetic performance.

Therapeutic targeting of TNFR2, which is selectively expressed on Tregs and certain immune subsets, represents a compelling strategy owing to its restricted expression profile. This selectivity underpins the considerable promise of TNFR2 agonism in treating autoimmune diseases by specifically expanding and activating immunosuppressive Tregs to restore immune homeostasis. Indeed, preclinical studies have demonstrated the potential efficacy of TNFR2 agonists in multiple autoimmune models (19, 20). A legitimate concern, however, is that excessive Treg expansion could, in theory, result in unintended immunosuppression, thereby compromising antitumor immunity or antiviral defense. It is therefore critical to emphasize that the disease context (e.g., autoimmunity versus cancer) and the dynamics of receptor activation (transient versus sustained) will be key determinants of safety. Future studies will aim to delineate these parameters to ensure an optimal balance between therapeutic efficacy and immune regulation for this promising strategy.

Future studies should focus on elucidating the detailed structural basis of peptide UMR2-705-TNFR2 interaction, evaluating its pharmacokinetic and pharmacodynamic properties, and assessing its efficacy in models of chronic inflammation and autoimmune pathology. Ultimately, the development of peptide UMR2–705 could open a new paradigm in targeted immunotherapy with improved specificity and tolerability.

## Materials and methods

### Mice and reagents

Female wild-type BALB/c and C57BL/6 mice (6–8 weeks old) were obtained from the Animal Facility of the University of Macau. All animal procedures were approved by and conducted in accordance with the guidelines of the Animal Research Ethics Committee of the University of Macau. Recombinant mouse interleukin-2 (IL-2), tumor necrosis factor (TNF), and anti-mouse monoclonal antibodies (CD45, TCR-β, CD4, Foxp3, CD8α, and TNFR2), as well as anti-human CD4 and Foxp3 antibodies, were purchased from BD Biosciences. Cytometric Bead Array (CBA) Mouse Inflammation Kits were also obtained from BD Biosciences. Lipopolysaccharide (LPS) was purchased from Sigma-Aldrich. LIVE/DEAD™ Fixable Near-IR Dead Cell Stain Kit, Foxp3/Transcription Factor Staining Buffer Set, FOXP3 antibodies, RPMI 1640 medium, penicillin-streptomycin solution, and fetal bovine serum (FBS) were acquired from Thermo Fisher Scientific. CD4 (L3T4) MicroBeads and LS Columns for magnetic cell sorting were obtained from Miltenyi Biotec.

### CD4+T cell purification and *in vitro* culture

CD4^+^ T cells were isolated from the spleen and lymph nodes of mice using CD4 (L3T4) MicroBeads (Miltenyi Biotec) according to the manufacturer’s protocol. Purified cells were seeded in U-bottom 96-well plates and cultured in RPMI 1640 medium supplemented with 10% fetal bovine serum (FBS), 2 mM L-glutamine, 10 mM HEPES, 0.1 mM non-essential amino acids, 1 mM sodium pyruvate, 1% penicillin (100 U/mL)/streptomycin (100 µg/mL), and 50 µM 2-mercaptoethanol. Cultures were maintained at 37°C in a humidified incubator with 5% CO_2_. To support CD4^+^ T cell viability, recombinant mouse IL-2 (10 ng/mL; BD Pharmingen) was added to the culture medium. For the induction of regulatory T cell (Treg) expansion and proliferation, recombinant mouse TNF (20 ng/mL; BD Pharmingen) was included.

### Isolation of human peripheral blood mononuclear cells

Peripheral blood was obtained from healthy donors through the Macao Blood Transfusion Center under an approved human subjects protocol. Whole blood was processed by density gradient centrifugation using Ficoll-Paque (Sigma) to isolate PBMCs. Cells collected at the interface were washed with phosphate-buffered saline (PBS).

### Generation, peptide treatment of BMDCs, and co-culture with T Cells

Bone marrow-derived dendritic cells (BMDCs) were generated from C57BL/6 mice. In brief, bone marrow cells were flushed from femurs and tibias. The cells were then cultured in RPMI-1640 medium supplemented with 10% FBS, 20 ng/mL recombinant murine GM-CSF (BD Pharmingen), and 10 ng/mL recombinant murine IL-4 (BD Pharmingen) for 7 days. Fresh medium containing cytokines was replenished on days 3 and 5. On day 7, immature BMDCs were harvested and in the presence or absence of peptide UMR2–705 for 24 hours.

The MACS-purified CD4^+^ T cells were then co-cultured with peptide UMR2-705-treated or control BMDCs at a ratio of 3:1 (T cells:BMDCs) in 96-well U-bottom plates for 72 hours. All cultures were maintained at 37°C in a 5% CO_2_ humidified incubator.

After co-culture, cells were harvested and analyzed by flow cytometry to determine the percentage of CD4^+^Foxp3^+^ regulatory T cells (Tregs). Intracellular staining for Foxp3 was performed using a Foxp3/Transcription Factor Staining Buffer Set according to standard protocols.

### RNA sequencing

Purified CD4^+^ T cells were cultured in RPMI 1640 complete medium supplemented with 10 ng/mL IL-2 and 20 ng/mL TNF, followed by treatment with either PBS or peptide UMR2-705. After 72 hours, total RNA was extracted from the cells. Library preparation and sequencing were performed by Shanghai Applied Protein Technology Co., Ltd. RNA sequencing libraries were constructed for Illumina according to the manufacturer’s protocol. Sequencing was conducted on an Illumina NovaSeq platform. Gene expression levels were quantified as fragments per kilobase of transcript per million mapped reads (FPKM).

### Inflammatory cytokine assay

Mouse serum levels of IL-6, TNF, and IL-17A were measured using the BD Cytometric Bead Array (CBA) Mouse Inflammation Kit, following the manufacturer’s instructions. Briefly, serum samples were incubated with capture bead sets, PE detection reagent, and PBS. The mixtures were incubated in the dark at room temperature for 2 hours. Serial dilutions of mouse inflammation standards were prepared to establish a standard curve. Following incubation, samples were analyzed by flow cytometry, and cytokine concentrations were determined by comparison to the standard curve.

### Synthesis and sequence of peptide

The peptide UMR2-705, with the amino acid sequence DLLISIY, was supplied by Nanjing TGpeptide Biotechnology Co., Ltd. It was synthesized employing standard solid-phase Fmoc (9-fluorenylmethoxycarbonyl) chemistry. Following synthesis, the crude peptide was purified to >95% purity by preparative reverse-phase high-performance liquid chromatography (RP-HPLC). The molecular weight and identity of the peptide were confirmed by mass spectrometry (MALDI-TOF or ESI-MS). The purified peptide was provided as a lyophilized powder and stored at -20°C until use.

### Flow cytometry

After blocking the Fc receptors, the cells were incubated with appropriately diluted antibodies. The cells were then suspended in FACS buffer for flow cytometry analysis. Data acquisition was performed via BD Fortessa and Celesta flow cytometers. Data analysis was conducted via FlowJo software (Tree Star Inc., Ashland, OR, USA).

### Molecular docking of peptide UMR2–705 to TNFR2

The three-dimensional structure of the extracellular domain of human TNFR2 (UniProt ID: P20333) was obtained from the RCSB Protein Data Bank (PDB ID: 3ALQ). The structure of peptide UMR2–705 was constructed and its geometry was optimized using energy minimization with the MMFF94 force field in Chem3D.

Molecular docking simulations were performed using AutoDock Vina 1.1.2 (46). The TNFR2 receptor structure was prepared by removing water molecules and adding polar hydrogen atoms and Kollman united atom charges. A grid box of sufficient size (e.g., 60 Å × 60 Å × 60 Å) was centered on the known TNF-binding site to encompass the entire receptor binding interface. The exhaustiveness parameter was set to 20 to ensure comprehensive sampling of conformational space.

The docking pose with the most favorable (lowest) binding energy (calculated to be -5.3 kcal/mol) was selected for further analysis. The protein-ligand interactions, including hydrogen bonds and hydrophobic contacts, were visualized and analyzed using Biovia Discovery Studio Visualizer or PyMOL (47).

### Statistical analysis

Data were analyzed using GraphPad Prism software (version 10). Comparisons among multiple groups were performed using one-way analysis of variance (ANOVA) followed by appropriate *post hoc* tests. Results are expressed as mean ± SEM from multiple independent experiments.

## Data Availability

The original contributions presented in the study are publicly available. This data can be found here: https://doi.org/10.6084/m9.figshare.30570512.v1.

## References

[1] Sakaguchi S Yamaguchi T Nomura T Ono M . Regulatory T cells and immune tolerance. Cell. (2008) 133:775–87. doi: 10.1016/j.cell.2008.05.009, PMID: 18510923

[2] Perdigoto AL Chatenoud L Bluestone JA Herold KC . Inducing and administering tregs to treat human disease. Front Immunol. (2015) 6:654. doi: 10.3389/fimmu.2015.00654, PMID: 26834735 PMC4722090

[3] Sharabi A Tsokos MG Ding Y Malek TR Klatzmann D Tsokos GC . Regulatory T cells in the treatment of disease. Nat Rev Drug Discov. (2018) 17:823–44. doi: 10.1038/nrd.2018.148, PMID: 30310234

[4] Chen X Baumel M Mannel DN Howard OM Oppenheim JJ . Interaction of TNF with TNF receptor type 2 promotes expansion and function of mouse CD4+CD25+ T regulatory cells. J Immunol. (2007) 179:154–61. doi: 10.4049/jimmunol.179.1.154, PMID: 17579033

[5] Chen X Hamano R Subleski JJ Hurwitz AA Howard OM Oppenheim JJ . Expression of costimulatory TNFR2 induces resistance of CD4+FoxP3- conventional T cells to suppression by CD4+FoxP3+ regulatory T cells. J Immunol. (2010) 185:174–82. doi: 10.4049/jimmunol.0903548, PMID: 20525892 PMC6314668

[6] Chen X Wu X Zhou Q Howard OM Netea MG Oppenheim JJ . TNFR2 is critical for the stabilization of the CD4+Foxp3+ regulatory T. cell phenotype in the inflammatory environment. J Immunol. (2013) 190:1076–84. doi: 10.4049/jimmunol.1202659, PMID: 23277487 PMC3552130

[7] Chen X Oppenheim JJ . Therapy: Paradoxical effects of targeting TNF signalling in the treatment of autoimmunity. Nat Rev Rheumatol. (2016) 12:625–6. doi: 10.1038/nrrheum.2016.145, PMID: 27586383 PMC8502420

[8] Chen X Plebanski M . Editorial: the role of TNF-TNFR2 signal in immunosuppressive cells and its therapeutic implications. Front Immunol. (2019) 10:2126. doi: 10.3389/fimmu.2019.02126, PMID: 31555303 PMC6742683

[9] Zou H Li R Hu H Hu Y Chen X . Modulation of regulatory T cell activity by TNF receptor type II-targeting pharmacological agents. Front Immunol. (2018) 9:594. doi: 10.3389/fimmu.2018.00594, PMID: 29632537 PMC5879105

[10] Lamontain V Schmid T Weber-Steffens D Zeller D Jenei-Lanzl Z Wajant H . Stimulation of TNF receptor type 2 expands regulatory T cells and ameliorates established collagen-induced arthritis in mice. Cell Mol Immunol. (2019) 16:65–74. doi: 10.1038/cmi.2017.138, PMID: 29375132 PMC6318277

[11] Chopra M Biehl M Steinfatt T Brandl A Kums J Amich J . Exogenous TNFR2 activation protects from acute GvHD via host T reg cell expansion. J Exp Med. (2016) 213:1881–900. doi: 10.1084/jem.20151563, PMID: 27526711 PMC4995078

[12] Schmid T Falter L Weber S Muller N Molitor K Zeller D . Chronic inflammation increases the sensitivity of mouse treg for TNFR2 costimulation. Front Immunol. (2017) 8:1471. doi: 10.3389/fimmu.2017.01471, PMID: 29163535 PMC5681910

[13] Fischer R Sendetski M Del Rivero T Martinez GF Bracchi-Ricard V Swanson KA . TNFR2 promotes Treg-mediated recovery from neuropathic pain across sexes. Proc Natl Acad Sci U.S.A. (2019) 116:17045–50. doi: 10.1073/pnas.1902091116, PMID: 31391309 PMC6708347

[14] Fischer R Proske M Duffey M Stangl H Martinez GF Peters N . Selective activation of tumor necrosis factor receptor II induces antiinflammatory responses and alleviates experimental arthritis. Arthritis Rheumatol. (2018) 70:722–35. doi: 10.1002/art.40413, PMID: 29342501

[15] Okubo Y Mera T Wang L Faustman DL . Homogeneous expansion of human T-regulatory cells via tumor necrosis factor receptor 2. Sci Rep. (2013) 3:3153. doi: 10.1038/srep03153, PMID: 24193319 PMC3818650

[16] Torrey H Kuhtreiber WM Okubo Y Tran L Case K Zheng H . A novel TNFR2 agonist antibody expands highly potent regulatory T cells. Sci Signal. (2020) 13:eaba9600. doi: 10.1126/scisignal.aba9600, PMID: 33293464

[17] Ye C Brand D Zheng SG . Targeting IL-2: an unexpected effect in treating immunological diseases. Signal Transduct Target Ther. (2018) 3:2. doi: 10.1038/s41392-017-0002-5, PMID: 29527328 PMC5837126

[18] Muhammad S Fan T Hai Y Gao Y He J . Reigniting hope in cancer treatment: the promise and pitfalls of IL-2 and IL-2R targeting strategies. Mol Cancer. (2023) 22:121. doi: 10.1186/s12943-023-01826-7, PMID: 37516849 PMC10385932

[19] Chen Y Jiang M Chen X . Therapeutic potential of TNFR2 agonists: a mechanistic perspective. Front Immunol. (2023) 14:1209188. doi: 10.3389/fimmu.2023.1209188, PMID: 37662935 PMC10469862

[20] Faustman DL Davis M Kuhtreiber WM . TNFR2 agonism: basic science and promising treatment for multiple sclerosis and related diseases. Int J Mol Sci. (2025) 26:7839. doi: 10.3390/ijms26167839, PMID: 40869160 PMC12386426

[21] Fischer R Marsal J Gutta C Eisler SA Peters N Bethea JR . Novel strategies to mimic transmembrane tumor necrosis factor-dependent activation of tumor necrosis factor receptor 2. Sci Rep. (2017) 7:6607. doi: 10.1038/s41598-017-06993-4, PMID: 28747780 PMC5529482

[22] Fischer R Kontermann RE Pfizenmaier K . Selective targeting of TNF receptors as a novel therapeutic approach. Front Cell Dev Biol. (2020) 8:401. doi: 10.3389/fcell.2020.00401, PMID: 32528961 PMC7264106

[23] Kaspar AA Reichert JM . Future directions for peptide therapeutics development. Drug Discov Today. (2013) 18:807–17. doi: 10.1016/j.drudis.2013.05.011, PMID: 23726889

[24] Hamano R Huang J Yoshimura T Oppenheim JJ Chen X . TNF optimally activatives regulatory T cells by inducing TNF receptor superfamily members TNFR2, 4-1BB and OX40. Eur J Immunol. (2011) 41:2010–20. doi: 10.1002/eji.201041205, PMID: 21491419 PMC3783213

[25] Mukai Y Nakamura T Yoshikawa M Yoshioka Y Tsunoda S Nakagawa S . Solution of the structure of the TNF-TNFR2 complex. Sci Signal. (2010) 3:ra83. doi: 10.1126/scisignal.2000954, PMID: 21081755

[26] Mensink M Verleng LJ Schrama E Janssen GM Tjokrodirijo RT Van Veelen PA . Tregs from human blood differentiate into nonlymphoid tissue-resident effector cells upon TNFR2 costimulation. JCI Insight. (2024) 9(5):e172942. doi: 10.1172/jci.insight.172942, PMID: 38341270 PMC10972588

[27] Lubrano di Ricco M Ronin E Collares D Divoux J Gregoire S Wajant H . Tumor necrosis factor receptor family costimulation increases regulatory T-cell activation and function via NF-kappaB. Eur J Immunol. (2020) 50:972–85. doi: 10.1002/eji.201948393, PMID: 32012260 PMC7383872

[28] Vargas J Lang I Stepanzow S Zaitseva O Arellano-Viera E Graf C . TNFR2, GITR and DR3 agonists exert distinct response durations in treg-mediated acute graft-versus-host disease protection. Blood. (2024) 144:3397–7. doi: 10.1182/blood-2024-209659

[29] Bai J Ding B Li H . Targeting TNFR2 in cancer: all roads lead to Rome. Front Immunol. (2022) 13:844931. doi: 10.3389/fimmu.2022.844931, PMID: 35251045 PMC8891135

[30] Chen X Subleski JJ Kopf H Howard OM Mannel DN Oppenheim JJ . Cutting edge: expression of TNFR2 defines a maximally suppressive subset of mouse CD4+CD25+FoxP3+ T regulatory cells: applicability to tumor-infiltrating T regulatory cells. J Immunol. (2008) 180:6467–71. doi: 10.4049/jimmunol.180.10.6467, PMID: 18453563 PMC2699949

[31] Mårtensson L Kovacek M Holmkvist P Semmrich M Svensson C Blidberg T . 725 Pre-clinical development of TNFR2 ligand-blocking BI-1808 for cancer immunotherapy. BMJ Specialist J. (2020) 8(Suppl 3):A768–A768. doi: 10.1136/jitc-2020-SITC2020.0725

[32] Krishnan S Alvarado R Huang G Yang X Filbert EL . Abstract LB175: APX601, a potent TNFR2 antagonist as a novel and promising approach to reverse tumor immune suppression. Cancer Res. (2021) 81:LB175–5. doi: 10.1158/1538-7445.AM2021-LB175

[33] Chen Y Jia M Xu S Zhao Y Chan E Zhang M . AN3025: a novel anti-human TNFR2 antibody that exhibits immune activation and strong anti-tumor activity *in vivo*. Cancer Res. (2021) 81:1451–1. doi: 10.1158/1538-7445.AM2021-1451

[34] Sum CS Danton M Hu Q Pritsker A Lin R Yu R . Novel TNFR2 antibodies to overcome T cell exhaustion and suppressive tumor microenvironment. Cancer Res. (2021) 81:1869–9. doi: 10.1158/1538-7445.AM2021-1869

[35] Mårtensson L Cleary K Semmrich M Kovacek M Holmkvist P Svensson C . Targeting TNFR2 for cancer immunotherapy: Ligand blocking depletors versus receptor agonists. Cancer Res. (2020) 80:936. doi: 10.1158/1538-7445.AM2020-936

[36] Wei S Fulton R Lu Y-Y Zhang Q Zhou H Raue A . Mechanism of action and biomarker strategy for HFB200301, an anti-TNFR2 agonist antibody for the treatment of cancer. Cancer Res. (2021) 81:1883–3. doi: 10.1158/1538-7445.AM2021-1883

[37] Sampson JF Kurella VB Paragas V Kumar S Lulo JE Qiu JA . A novel human TNFR2 antibody (MM-401) modulates T cell responses in anti-cancer immunity. Cancer Res. (2019) 79:555. doi: 10.1158/1538-7445.AM2019-555

[38] Henninot A Collins JC Nuss JM . The current state of peptide drug discovery: back to the future? J Med Chem. (2018) 61:1382–414. doi: 10.1021/acs.jmedchem.7b00318, PMID: 28737935

[39] Quazi S . TNFR2 antagonist and agonist: a potential therapeutics in cancer immunotherapy. Med Oncol. (2022) 39:215. doi: 10.1007/s12032-022-01772-2, PMID: 36175687

[40] Yang S Wang J Brand DD Zheng SG . Role of TNF-TNF receptor 2 signal in regulatory T cells and its therapeutic implications. Front Immunol. (2018) 9:784. doi: 10.3389/fimmu.2018.00784, PMID: 29725328 PMC5916970

[41] Chen X Nie Y Xiao H Bian Z Scarzello AJ Song NY . TNFR2 expression by CD4 effector T cells is required to induce full-fledged experimental colitis. Sci Rep. (2016) 6:32834. doi: 10.1038/srep32834, PMID: 27601345 PMC5013387

[42] Mensink M Tran TNM Zaal EA Schrama E Berkers CR Borst J . TNFR2 costimulation differentially impacts regulatory and conventional CD4(+) T-cell metabolism. Front Immunol. (2022) 13:881166. doi: 10.3389/fimmu.2022.881166, PMID: 35844585 PMC9282886

[43] Tam EM Fulton RB Sampson JF Muda M Camblin A Richards J . Antibody-mediated targeting of TNFR2 activates CD8(+) T cells in mice and promotes antitumor immunity. Sci Transl Med. (2019) 11(512):eaax0720. doi: 10.1126/scitranslmed.aax0720, PMID: 31578241

[44] Kim EY Teh SJ Yang J Chow MT Teh HS . TNFR2-deficient memory CD8 T cells provide superior protection against tumor cell growth. J Immunol. (2009) 183:6051–7. doi: 10.4049/jimmunol.0803482, PMID: 19841176

[45] Ye LL Wei XS Zhang M Niu YR Zhou Q . The significance of tumor necrosis factor receptor type II in CD8(+) regulatory T cells and CD8(+) effector T cells. Front Immunol. (2018) 9:583. doi: 10.3389/fimmu.2018.00583, PMID: 29623079 PMC5874323

[46] Trott O Olson AJ . AutoDock Vina: improving the speed and accuracy of docking with a new scoring function, efficient optimization, and multithreading. J Comput Chem. (2010) 31:455–61. doi: 10.1002/jcc.21334, PMID: 19499576 PMC3041641

[47] Schrodinger LLC . The axPyMOL Molecular Graphics Plugin for Microsoft PowerPoint, Version 1.8. New York City, NY. (2015).

