# Supplementary material for: A novel TNFR2 agonist peptide for the expansion of CD4+Foxp3+ regulatory T cells
Source: Front Immunol. 2025 Nov 26;16:1696587. doi: 10.3389/fimmu.2025.1696587 (PMC12689330; doi:10.3389/fimmu.2025.1696587)
Supplement: Supplementary file 1 [file DataSheet1.docx]

**Supplementary**


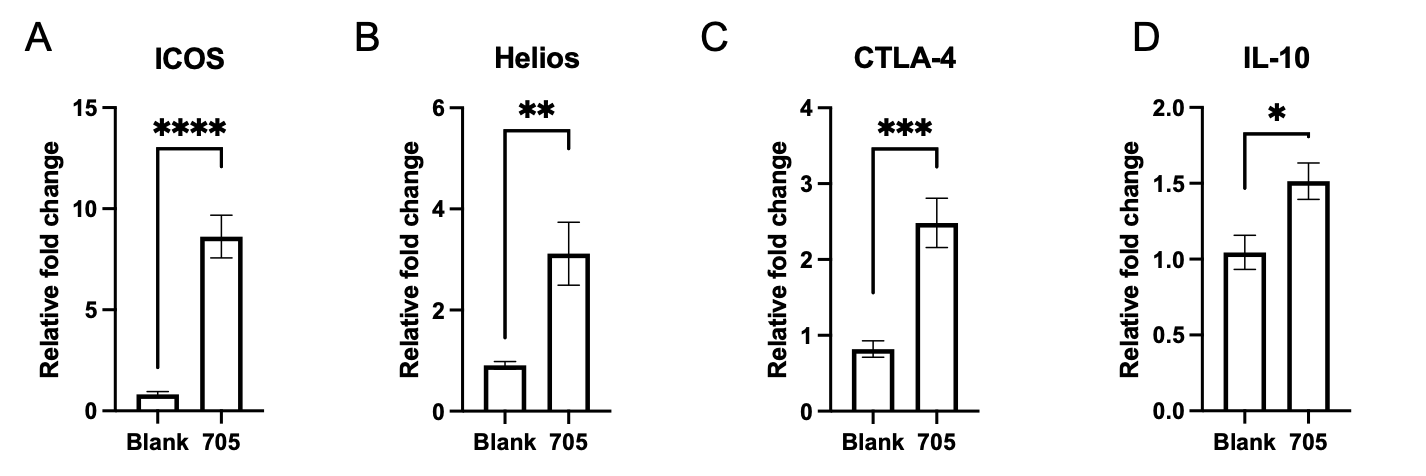


**Figure S1.** mRNA expression levels of suppressive markers in Treg cells after three days of UMR2-705 treatment. (A) ICOS, (B) Helios, (C) CTLA-4, and (D) IL-10.


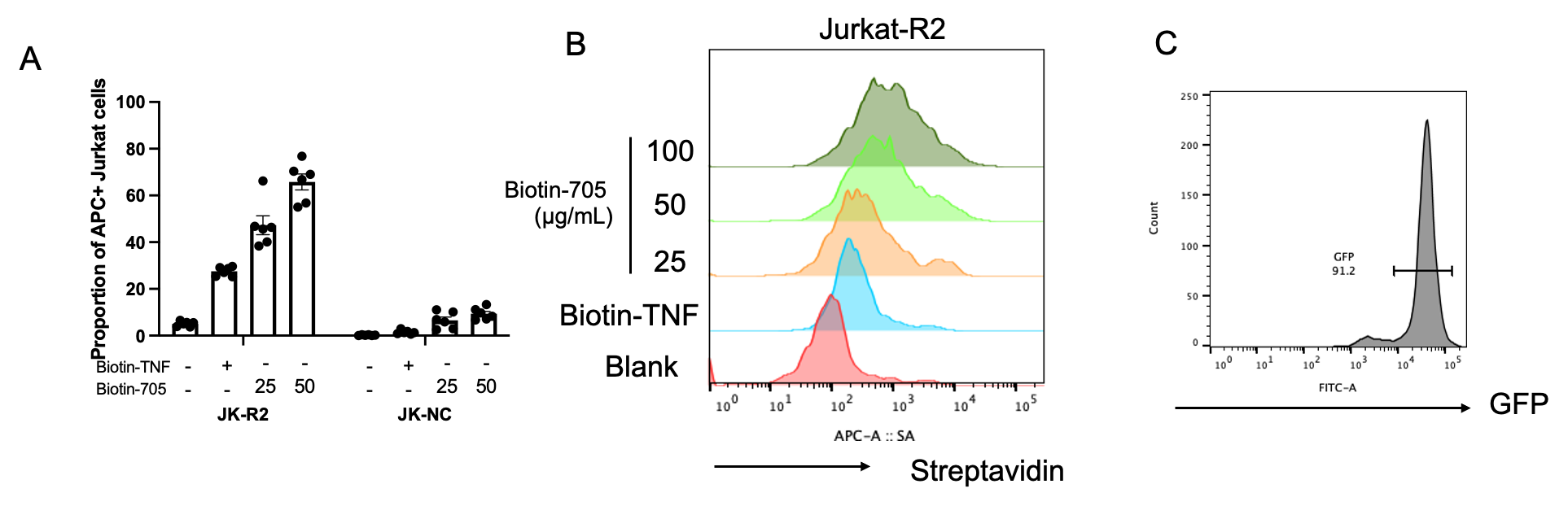


**Figure S2.** Detection of biotin-UMR2-705 binding on the surface of TNFR2-overexpressing Jurkat cells. TNFR2-overexpressing Jurkat cells (JK-R2) and control cells (JK-NC) were incubated with biotin-UMR2-705, followed by staining with APC-conjugated streptavidin to assess surface binding. (A) Flow cytometric analysis of biotin-UMR2-705 binding on JK-R2 and JK-NC cells. (B) Representative flow cytometry histogram illustrating biotin-UMR2-705 binding on the surface of Jurkat-R2 cells. (C) GFP fluorescence indicating successful transfection of TNFR2 in Jurkat cells.


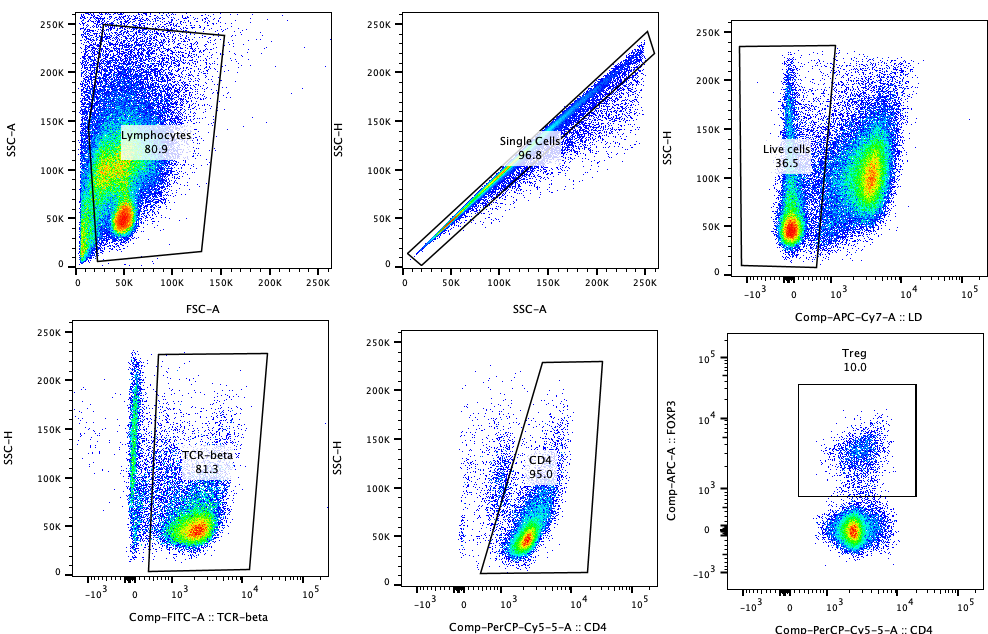


Figure S3. Gating strategy for CD4+ Foxp3+ Treg cells.

| Sample ID | Clone No. | TNFRSF1B protein | negetive control hFC | Peptide sequence |
| --- | --- | --- | --- | --- |
|  |  | OD450nm | OD450nm |  |
| 701 | 1 | 0. 8538 | 0. 0577 | WSLGYTG |
|  | 2 | 0. 8259 | 0. 0746 | WSLGYTG |
|  | 4 | 2. 0488 | 0. 1016 | WSLGYTG |
|  | 8 | 1.3324 | 0. 0599 | WSLGYTG |
|  | 10 | 1.3229 | 0.0471 | WSLGYTG |
|  | 14 | 2.232 | 0. 0752 | WSLGYTG |
|  | 20 | 1.1245 | 0. 0728 | WSLGYTG |
| 702 | 12 | 1. 6919 | 0. 0665 | WSLGYPG |
| 703 | 3 | 1. 3281 | 0. 0631 | WSFGHSG |
| 704 | 6 | 0. 8645 | 0. 0659 | WSSGHSG |
| 705 | 7 | 1.9781 | 0. 0654 | DLLISIY |
| 706 | 9 | 1.3373 | 0. 0587 | LGGMVHT |
| 707 | 11 | 1.5642 | 0. 0672 | SFPARVI |
| 708 | 15 | 1.9167 | 0. 0566 | MLPFPGS |
| 709 | 16 | 1. 3997 | 0. 0833 | AHSTVMW |
| 710 | 17 | 2. 1915 | 0. 0827 | SLNHTLI |
| 711 | 18 | 1.3257 | 0. 0637 | SNTAWNK |
| 712 | 19 | 1. 5933 | 0. 0731 | TFHPLLF |
| 713 | 13 | 2.5103 | 0. 0616 | RSLGYPG |

**Supplementary Table 1.** ELISA-based detection of binding activity between individual phage-displayed peptide clones and TNFR2 protein after the third round of biopanning. The table shows the OD₄₅₀ values representing the relative binding affinities of each clone to TNFR2.
